# Saddle Pulmonary Embolism in a Patient With Chronic Kidney Disease and Gastric Malignancy: A Case Report

**DOI:** 10.1155/carm/8832129

**Published:** 2025-12-11

**Authors:** Abigayle Therese R. Guiritan, Oscar D. Naidas, Charles Patrick D. Uy

**Affiliations:** ^1^ Department of Medicine, St. Luke’s Medical Center, Quezon City, Philippines, stluke.com.ph

**Keywords:** chronic kidney disease, erythropoietin use, gastric adenocarcinoma, saddle pulmonary embolism

## Abstract

**Background:**

Anemia of chronic renal disease as well as cancer and chemotherapy‐induced anemia (CIA) are often associated with poor outcomes, and the use of erythropoietin stimulating agents (ESAs) for patients with chronic kidney disease (CKD) and anemia associated with cancer has been a common practice. However, the increased incidence of venous thromboembolism has been reported in these populations.

**Clinical Presentation:**

A 61‐year‐old male, known case of CKD stage 3A secondary to hypertension nephrosclerosis since 2019, diagnosed case of gastric adenocarcinoma, poorly differentiated with signet ring features, proximal corpus, stage IV (2022) s/p port‐a‐catheter insertion, s/p cycle 4 leucovorin calcium (folinic acid), fluorouracil, and oxaliplatin (FOLFOX) with nivolumab for palliative chemotherapy, and anemia multifactorial from chronic disease, with chemotherapy use maintained on erythropoietin beta 10,000 IU once weekly for 3 months with hemoglobin ranges from 8.1 to 12 g/dL came in for cycle 5 chemotherapy. On review of systems, the patient complained of dry cough mostly in the evening accompanied by exertional dyspnea. A 12‐L ECG revealed sinus rhythm with S1Q3T3 pattern. The 2D echo with Doppler revealed a dilated right ventricle with hypocontractile walls with fractional area change of 17% with moderate pulmonary hypertension (pulmonary artery systolic pressure of 52.4 mmHg). D‐dimer was elevated at 17,290. Enoxaparin 0.8 mL (1 mg/kg/bid) subcutaneously every 12 h was started, and erythropoietin beta was discontinued. On the second hospital day, he had persistent coughing episodes accompanied by desaturation as low as 88% at room air. Hence, the patient was given oxygen supplementation at 2lpm nasal cannula and started with piperacillin tazobactam to treat for pneumonia. Within the day, he developed hypotension as low as 80/60 mmHg, and he was hooked to norepinephrine drip initially at 0.05 mcg/kg/min. The venous compression test showed acute extensive proximal deep venous thrombosis (DVT), totally occluding the left common femoral, proximal to distal femoral and popliteal veins, and acute distal DVT, totally occluding the right soleal vein. CTPA confirmed the presence of saddle pulmonary embolism (PE). Enoxaparin was shifted to unfractionated heparin 5000 IU as bolus and then started on heparin drip, and he was transferred to the intensive care unit. Thrombolysis with alteplase 100 mg intravenously and repeat 2D echo was done, which now revealed normal pulmonary artery systolic pressure from 52.4 mmHg to 26.5 mmHg by tricuspid regurgitant jet method with improvement of fractional area change to 28.4% from 17%. Enoxaparin 0.8 mL subcutaneously every 12 h was resumed. However, he was beginning inter/intramuscular hematoma formation on the right upper back was noted. Anticoagulation was temporarily put on hold and on the fifth hospital day, he underwent IVC filter insertion for extensive acute lower limb thrombosis. Khorana score was 3 (gastric malignancy + hemoglobin level < 10 g/dL or using RBC growth factors), which was high risk for venous thromboembolism. Hence, he was discharged with enoxaparin 0.6 mL subcutaneously twice daily.

**Conclusion:**

Although with clinical benefits, the use of erythropoietin is still individualized, especially in patients with CKD and malignancy. Recognition of the multiple factors that may predispose a patient to develop PE is important for prompt intervention, which could improve patient outcomes.

## 1. Introduction

Anemia of chronic renal disease as well as cancer and chemotherapy‐induced anemia (CIA) are often associated with poor outcomes. It may have a negative impact on the quality of life and may influence the efficacy of treatment, disease progression, and even survival [1]. Historically, erythropoietin has been known as the primary hematopoietic growth factor which regulates cellular proliferation and differentiation of the erythroid lineage, leading to frequent usage of erythropoietin stimulating agents (ESAs) for patients with chronic kidney disease (CKD) and anemia associated with cancer [2]. It is currently approved for the treatment of patients with symptomatic anemia caused by palliative chemotherapy to reduce the number of RBC transfusions and gradually improve anemia‐related symptoms [3]. Despite its clinical benefit, current management options for anemia in CKD are controversial, with some clinical trials indicating raised morbidity and mortality associated with erythropoiesis‐stimulating agents [4]. In CIA, both randomized clinical studies and systematic reviews demonstrated a significantly higher risk of thromboembolic events in patients receiving ESAs for CIA than that in the placebo groups [5, 6]. This is a case of a massive pulmonary embolism (PE) in a 61‐year‐old male patient with CKD and stage IV gastric adenocarcinoma.

## 2. Case Presentation

**Figure 1:**
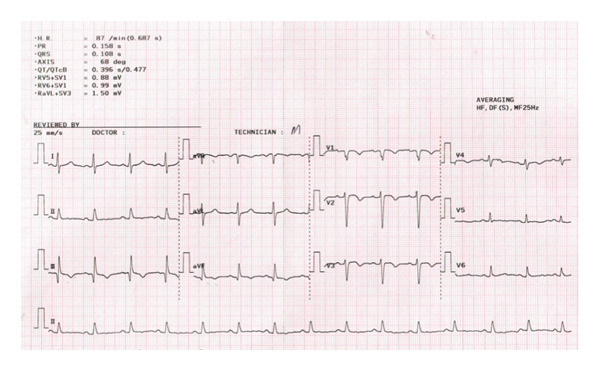
12‐L ECG showing S1Q3T3 pattern, sinus rhythm, and poor R wave progression.

**Figure 2:**
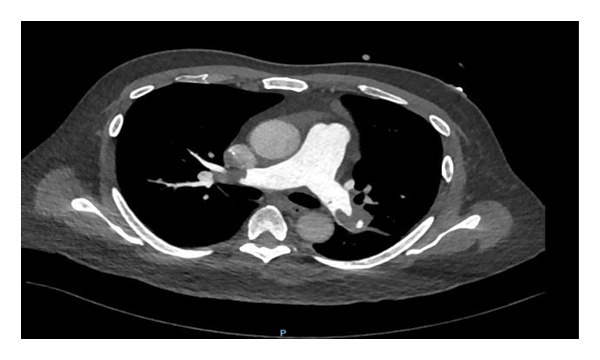
CTPA revealing saddle pulmonary embolism.

**Figure 3:**
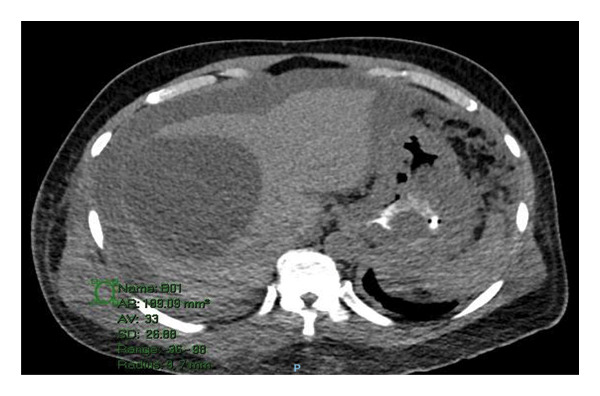
CT scan finding of a thin‐walled fluid collection is better seen in the region of the right upper paraspinal and trapezius muscles having a dimension of about 3.3 × 7.4 × 3.7 cm (AP × T × CC). Some hyperdense components are also seen. Minimal fluid collection is also noted underneath the right serratus anterior. Subcutaneous and myofascial edema are noted in both sides of the chest, but mostly on the right.

**Figure 4:**
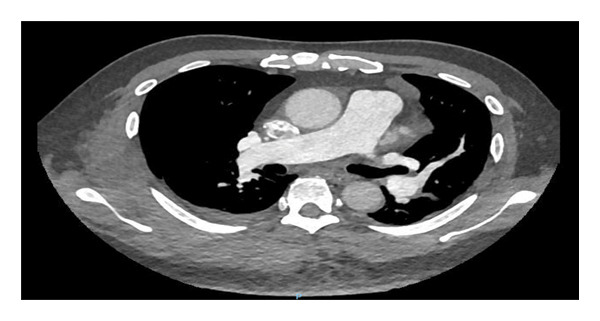
Repeat CTPA showing a decrease in the size of the saddle pulmonary embolus.

A 61‐year‐old male, known case of CKD stage 3A secondary to hypertension nephrosclerosis since 2019, recently diagnosed last 2022 with gastric adenocarcinoma, poorly differentiated with signet ring features, proximal corpus, stage IV s/p port‐a‐catheter insertion, s/p cycle 4 leucovorin calcium (folinic acid), fluorouracil, and oxaliplatin (FOLFOX) with nivolumab for palliative chemotherapy, and anemia multifactorial from chronic disease, with chemotherapy use maintained on erythropoietin beta 10,000 IU once weekly for 3 months with hemoglobin ranges from 8.1 to 12 g/dL came in for cycle 5 chemotherapy. On review of systems, the patient complained of dry cough mostly in the evening accompanied by exertional dyspnea. He was prescribed with antitussive medication but with minimal relief and hence referred to pulmonary and cardiology services for workup. On assessment, he had stable vital signs with no desaturation at room air. He had no neck vein distention, with clear breath sounds, normal rate and regular rhythm, and full pulses with grade 1 bipedal edema. A 12‐L ECG revealed sinus rhythm with S1Q3T3 pattern (Figure 1). The 2D echo with Doppler revealed a dilated right ventricle with hypocontractile walls with fractional area change of 17% with moderate pulmonary hypertension (pulmonary artery systolic pressure of 52.4 mmHg). Wells score was computed, which revealed moderate risk (PE is primary diagnosis or equally likely, heart rate of > 100 beats per minute, and malignancy with treatment within 6 months). The venous compression test showed acute extensive proximal deep venous thrombosis (DVT), totally occluding the left common femoral, proximal to distal femoral and popliteal veins, and acute distal DVT, totally occluding the right soleal vein. D‐dimer was elevated at 17,290. Enoxaparin 0.8 mL (1 mg/kg/bid) subcutaneously every 12 h was started, erythropoietin beta was discontinued, and he was scheduled for CT pulmonary angiogram (CTPA) and was transferred to progressive care unit. On the second hospital day, he had persistent coughing episodes accompanied by desaturation as low as 88% at room air. Hence, he was given oxygen supplementation at 2lpm nasal cannula and started with piperacillin tazobactam to treat for pneumonia. However, on the same day, he developed hypotension as low as 80/60 mmHg. Fluid resuscitation with PNSS 500 mL was done but he remained to be hypotensive and was hooked to norepinephrine drip initially at 0.05 mcg/kg/min. CTPA showed saddle PE (Figure 2). Enoxaparin was shifted to unfractionated heparin 5000 IU as bolus and then started on heparin drip and was transferred to the intensive care unit. Thrombolysis with alteplase 100 mg intravenously and repeat 2D echo was done, which now revealed normal pulmonary artery systolic pressure from 52.4 mmHg to 26.5 mmHg by the tricuspid regurgitant jet method with improvement of fractional area change to 28.4% from 17%. Enoxaparin 0.8 mL subcutaneously every 12 h was resumed. However, he had beginning inter/intramuscular hematoma formation on the right upper back (Figure 3). Anticoagulation was temporarily put on hold and on the fifth hospital day, patient underwent inferior vena cava (IVC) filter insertion for extensive acute lower limb thrombosis. Repeat CTPA was done, showing moderate to marked regression of the bilateral pulmonary emboli (Figure 4). After clearance from all services, he was able to undergo cycle 5 of chemotherapy. He remained to be stable and was eventually discharged on the 15th hospital day. Khorana score was 3 (gastric malignancy + hemoglobin level < 10 g/dL or using RBC growth factors), which was high risk for venous thromboembolism. Hence, he was discharged with enoxaparin 0.6 mL subcutaneously twice daily. On follow‐up, a total of 16 cycles of FOLFOX + 13 sessions of nivolumab were done. However, disease continued to progress. Chemotherapy was shifted to Docetaxel + Carboplatin with 5 fluorouracil. Patient eventually succumbed to death due to pneumonia.

## 3. Discussion

Saddle PE is a rare type of acute PE, accounting for 2.6%–5.4% of all acute PE cases, which presents as a visible thrombus located at the bifurcation of the main pulmonary artery that can lead to sudden hemodynamic collapse and death [7]. Risk factors for venous thromboembolism are multifactorial and include major surgery, trauma, cancer, obesity, diabetes, use of implantable venous access device, and hereditary predisposition [8]. Although not included in the common risk factors for VTE, Wattanakit et al. suggested that middle‐aged and elderly patients with CKD (stages 3 through 4) are at increased risk for incident VTE [9].

Data from the Overview of Prospective Investigation of Pulmonary Embolism Diagnosis II (PIOPED II) study revealed that new dyspnea at rest or on exertion was the most frequent symptom in patients with PE in general and no prior cardiopulmonary disease (73%) [10]. Some of the predictors of worse outcome in VTE include massive PE, malignancy, and CKD [11]. In a study done by Singh et al. [12], PE patients with CKD died 1.15 times more often than those with normal kidney function and PE patients with ESRD died 4.2 times more often than those with normal kidney function. Although some studies claim an increase incidence of venous thromboembolism with erythropoietin use [13, 14], systematic review and network meta‐analysis done by Zheng et al. revealed no statistical differences or closed links between node networks on erythropoietin use and the incidence of deep vein thrombosis and PE [15].

High‐risk PE which is defined as hemodynamic instability from PE is a complex, life‐threatening condition, and emergency clinicians must be ready to resuscitate and rapidly pursue primary reperfusion therapy [16]. Primary management for patients with high‐risk PE is the use of systemic thrombolytics like unfractionated heparin, which was given to our patient. Hemodynamic instability was addressed with the use of norepinephrine, which causes vasoconstriction to support blood pressure without disproportionately increasing pulmonary vascular resistance (PVR). Oxygen supplementation with nasal cannula was also administered to improve oxygenation without increasing intrathoracic pressure. IVC filters are used to prevent PE in patients with contraindications to, complications of, or failure of anticoagulation therapy and patients with extensive free‐floating thrombi or residual thrombi following massive PE [17]. The patient developed inter/intramuscular hematoma formation on the right upper back, hence a decision was made to temporarily hold anticoagulation and an IVC filter was inserted due to the presence of extensive acute lower limb thrombosis.

Our patient was a known CKD on erythropoietin 10,000 IU subcutaneously once a week for 3 months and had an active malignancy of stage IV gastric adenocarcinoma who developed a massive saddle PE.

## 4. Conclusion

Although with clinical benefits, the use of erythropoietin is still individualized, especially in patients with CKD and malignancy. Recognition of the multiple factors that may predispose a patient to develop PE is important for prompt intervention, which could improve patient outcomes.

## Consent

The patient allowed personal data processing and informed consent was obtained to be included in the study.

## Conflicts of Interest

The authors declare no conflicts of interest.

## Funding

No funding was received for this manuscript.

## Data Availability

The data that support the findings of this study are available on request from the corresponding author. The data are not publicly available due to privacy or ethical restrictions.
